# The influence of adolescent nicotine exposure on ethanol intake and brain gene expression

**DOI:** 10.1371/journal.pone.0198935

**Published:** 2018-06-18

**Authors:** Constanza P. Silva, William J. Horton, Michael J. Caruso, Aswathy Sebastian, Laura C. Klein, Istvan Albert, Helen M. Kamens

**Affiliations:** 1 Biobehavioral Health Department, Pennsylvania State University, University Park, Pennsylvania, United States of America; 2 Department of Animal Science, Pennsylvania State University, University Park, Pennsylvania, United States of America; 3 Biochemistry and Molecular Biology, Pennsylvania State University, University Park, Pennsylvania, United States of America; Oregon Health and Science University, UNITED STATES

## Abstract

Nicotine and alcohol are often co-abused. Adolescence is a vulnerable period for the initiation of both nicotine and alcohol use, which can lead to subsequent neurodevelopmental and behavioral alterations. It is possible that during this vulnerable period, use of one drug leads to neurobiological alterations that affect subsequent consumption of the other drug. The aim of the present study was to determine the effect of nicotine exposure during adolescence on ethanol intake, and the effect of these substances on brain gene expression. Forty-three adolescent female C57BL/6J mice were assigned to four groups. In the first phase of the experiment, adolescent mice (PND 36–41 days) were exposed to three bottles filled with water or nicotine (200 μg/ml) for 22 h a day and a single bottle of water 2 h a day for six days. In the second phase (PND 42–45 days), the 4-day Drinking-in-the-Dark paradigm consisting of access to 20% v/v ethanol or water for 2h or 4h (the last day) was overlaid during the time when the mice did not have nicotine available. Ethanol consumption (g/kg) and blood ethanol concentrations (BEC, mg %) were measured on the final day and whole brains including the cerebellum, were dissected for RNA sequencing. Differentially expressed genes (DEG) were detected with CuffDiff and gene networks were built using WGCNA. Prior nicotine exposure increased ethanol consumption and resulting BEC. Significant DEG and biological pathways found in the group exposed to both nicotine and ethanol included genes important in stress-related neuropeptide signaling, hypothalamic–pituitary–adrenal (HPA) axis activity, glutamate release, GABA signaling, and dopamine release. These results replicate our earlier findings that nicotine exposure during adolescence increases ethanol consumption and extends this work by examining gene expression differences which could mediate these behavioral effects.

## 1. Introduction

Nicotine and ethanol are often used concomitantly. Smoking rates among alcoholics are estimated to be higher than in the general population (around 80% vs. 34%) and the prevalence of alcoholism in the United States has been calculated to be 10 times higher in smokers than among non-smokers [1,2]. Adolescence is a vulnerable period for the onset of nicotine and ethanol use [3–5], with evidence linking the risk of smoking during this period with subsequent development of alcohol abuse and dependence [6–8]. Additionally, sex differences have been reported, suggesting a stronger association between concurrent smoking and alcohol use disorders (AUDs) among females as compared to males [9–12]. Because the majority of smokers begin smoking during adolescence [13], these findings suggest that adolescent females may be especially vulnerable to negative consequences of early alcohol and tobacco use.

Nicotine exposure during adolescence has unique effects on the developing brain. Exposure to nicotine during this period produces long-term alterations in developing structures such as the neocortex, hippocampus, and cerebellum [14]. Nicotine alters the function of these brain regions by inducing changes in dendritic spines and neuronal morphology, that are produced by alterations in transcriptional regulators of synapse maintenance [15]. These nicotine-induced neurobiological alterations can produce cognitive impairment, increase risk-taking behaviors [16], and increase risk of future depression [17] or anxiety [18]. These biological and behavioral alterations can predispose certain individuals to develop substance use disorders [19]. Therefore, it is crucial to examine the effects of adolescent nicotine exposure on physiological and behavioral outcomes. One such physiological response is changes in gene expression. These changes could alter normal developmental trajectories, increasing the risk of substance use later in life.

In animal models, age and sex-related differences in nicotine and ethanol consumption have been identified. Adolescent rodents show age-related differences in nicotine sensitivity, reward, tolerance, withdrawal, and nicotinic acetylcholine receptor (nAChRs) function compared to adults [20–23]. Further, adolescent female mice and rats consume more nicotine (adjusted for body weight) than do their male counterparts [24–26]. Moreover, adolescent female mice are more responsive to the rewarding effects of nicotine [27] and more susceptible to binge ethanol drinking compared to males [28,29].

Previous studies have shown that nicotine exposure increases ethanol self-administration in rodents [30,31]. One proposed mechanism by which nicotine increases ethanol self-administration is via the release of stress hormones [32–33]. Nicotine activates the stress-responsive neuroendocrine system (i.e. hypothalamic–pituitary–adrenal (HPA)) and, consequently, induces glucocorticoid release [32]. In adult rodents, glucocorticoids reduce ethanol-induced dopamine signaling through enhancement of GABAergic inhibition on dopamine (DA) neurons in the ventral tegmental area (VTA) [31,34,35]. Further, blunted DA levels have been associated with increased susceptibility to drug and ethanol use [36]. Ethanol can also potentiate GABA_A_, nACh, and 5-HT3 receptor function, and inhibit the function of glutamatergic receptors [37]. However, these studies in adult animals have focused on either nicotine or ethanol’s specific mechanisms of action rather than the effects of these substances on adolescent brain development and their link to later drug behaviors.

The effect of adolescent nicotine exposure on brain gene expression and ethanol consumption are poorly understood. The aim of this study was to determine the effect of nicotine exposure on ethanol consumption and resulting gene expression in female adolescent C57BL/6J mice. Our findings reveal that nicotine exposure increases ethanol consumption and blood ethanol concentrations (BEC) in female adolescent mice compared to nicotine-naïve animals. Significant differentially expressed genes (DEG) and biological pathways after nicotine and/or ethanol administration were associated with neuropeptide, HPA axis activity, neurogenesis, glutamatergic and GABAergic neurotransmission, and DA release. Our results allow us to hypothesize that nicotine exposure alters stress-related neuroendocrine and reward-associated neurotransmitter systems, which may mediate enhanced ethanol consumption, however, future work is required to test this.

## 2. Materials and methods

### 2.1. Animals

Forty-three adolescent (PND 28) female C57BL/6J mice were purchased from The Jackson Laboratory, Bar Harbor, ME. Only female mice were tested due to reported differences in nicotine consumption and ethanol effects observed between sexes [26,38–40]. Mice were singly housed in standard sized Plexiglas cages with bedding (Bed-o’Cobs, The Anderson Agriservices, Inc. Maume, OH) in a temperature-controlled room (20.3°C ± 0.8). Animals were housed on a 12-hour reversed light/dark cycle (lights off at 1000 h). Mice had *ad libitum* food (Lab Rodent Diet 5001, PMI Nutrition International, Inc., Brentwood, MO) throughout the experiment. All procedures were approved by the Pennsylvania State University Institutional Animal Care and Use Committee (Protocol Number: 45610).

### 2.2. Behavioral paradigm

#### 2.2.1. Baseline

**Fig 1:**
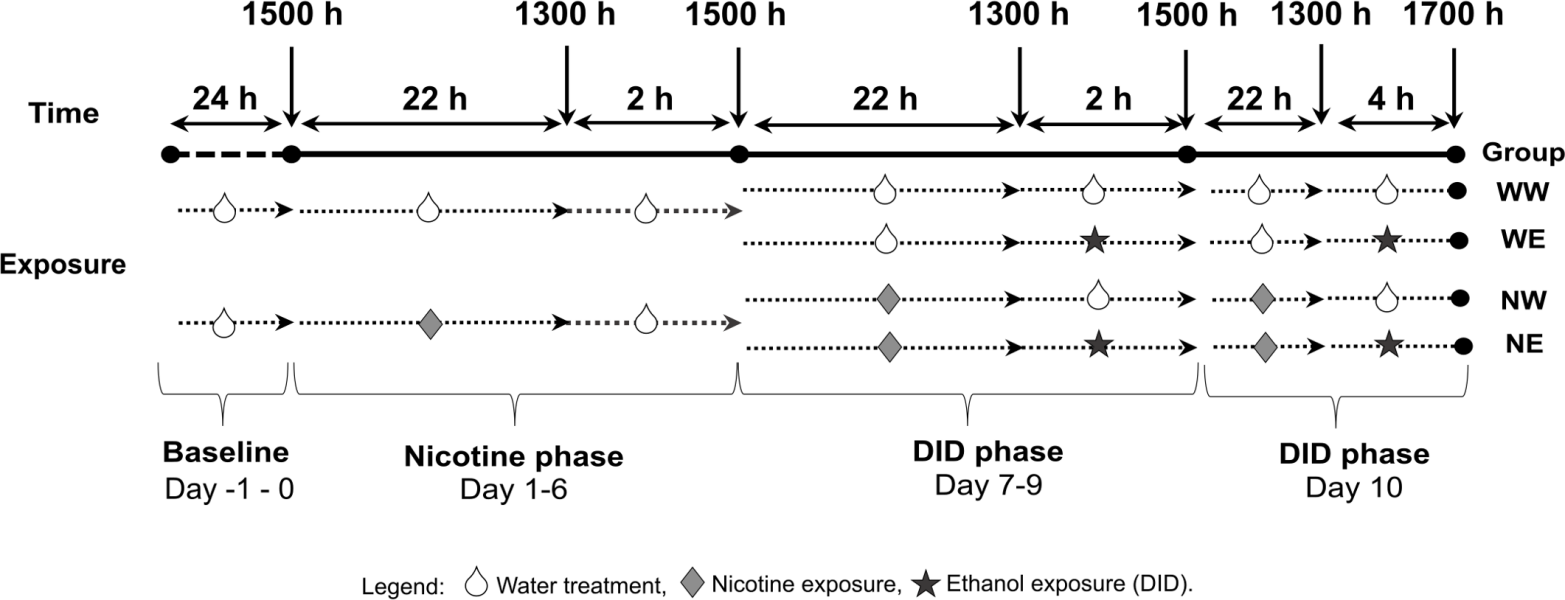
Experimental timeline.

During the baseline period (PND 33–35; Fig 1), mice had 24 h access to tap water in a single drinking bottle. Body weight and fluid consumption were measured daily.

#### 2.2.2. Nicotine treatment

Mice were randomly assigned into four groups: Water-Water (WW), Water-Ethanol (WE), Nicotine-Water (NW), or Nicotine-Ethanol (NE). A WW and NW group were included to control for the effects of nicotine on overall thirst. There were 10–12 mice per group. During the first six days of the experiment, mice were exposed to 3 glass drinking bottles filled with water or nicotine for 22 h a day, and a single water bottle for 2 h each day (Fig 1). For the WW and WE groups, all 3 bottles were filled with tap water. For the NW and NE groups, all 3 bottles were filled with 200 μg/ml (−)-nicotine freebase (Sigma–Aldrich, St. Louis, MO) dissolved in tap water. This concentration of nicotine was chosen because it is voluntary consumed by adolescent mice without adverse effects [25,41,42] and replicates our prior work [35]. Bottles were placed on the cages at 1500 h and were removed and replaced with the single water bottle at 1300 h the next day. The three bottles were read and nicotine consumption (mg/kg) was calculated for each mouse. The 2 h single bottle was weighed and water consumption (ml) was calculated. Leakage/evaporation was accounted for by tubes on control cages handled using the same protocol, but with no animal present. We subtracted the volume lost in control tubes from individual drinking values. These procedures continued throughout the experiment. However, during the last 4 days (PND 42–45) mice were exposed to ethanol via the drinking-in-the-dark (DID) protocol (see section 2.2.3).

#### 2.2.3. Drinking-in-the-dark (DID) protocol

The DID protocol was performed as previously reported [40,43]. During experimental days (7–10) nicotine exposure continued as detailed above (22 h/day), however, at 1300 h, all 3 bottles (nicotine or water) were removed and replaced with a single 10 ml serological pipette fitted with a ball bearing drinking spout containing either ethanol or water. Ethanol was prepared from ethyl alcohol (200 proof; Koptec 200) diluted in tap water to produce a 20% v/v solution [43,44]. Mice had 2 h access to a single bottle of water or ethanol for three days (PND 42–44). On the final day (PND 45), mice had 4 h access to the ethanol bottle. Leakage/evaporation was accounted for by tubes on control cages as described above. At the end of the 4 h drinking session on the final day, blood samples were collected from the tail vein (10μl) and mice were sacrificed via cervical dislocation, whole brains including the cerebellum were dissected and placed into RNAlater® for subsequent RNA extraction.

### 2.3. Blood ethanol concentration (BEC) assessment

BEC were examined with an enzymatic assay [45–47]. This assay links the conversion of ethanol to acetaldehyde together with the conversion of NAD to NADH by the addition of alcohol dehydrogenase (ADH). NADH production was quantified with a spectrophotometer (340nm). Individual BEC values were determined using a standard curve run in parallel with the samples [47].

### 2.4. Statistical analysis of behavioral data

Nicotine, ethanol, and water consumption as well as BEC were dependent variables. Group and experimental day were used as independent factors. A repeated measure ANOVA was performed to analyze nicotine consumption throughout the experiment, followed by a Tukey’s *post hoc* test (day 6 was not analyzed because of missing data). Based on our previous results [35], a one-tailed *t-test* was conducted to analyze differences in BEC and ethanol consumption between groups with alpha set at 0.05 because we predicted that nicotine would increase ethanol consumption. These analyses were conducted in Statistical Program for Social Sciences (SPSS, Chicago, IL) or in R (version 3.2.2, R Core Team, 2015).

### 2.5. RNA extraction

RNA was extracted from a randomly selected subset of mice (16 total; 4 samples from each experimental group). Total RNA was extracted with an RNeasy® Midi Kit (QIAGEN, Valencia, CA). RNA quality was assessed using an Agilent 2100 BioAnalyzer™ (Agilent Technologies, Santa Clara, CA). RNA Integrity Number (RIN) was on average 8.23 ± 0.26 for all samples, suggesting high RNA integrity and quality [48].

### 2.6. Library preparation RNA-sequencing

An Illumina TruSeq® Stranded mRNA Library Prep Kit (Illumina, San Diego, CA) was used for cDNA library preparation following the manufacturers’ protocol [49]. An Agilent 2100 BioAnalyzer^TM^ was used for library sizing, cDNA quantification, and quality measurement. Finally, libraries were sequenced using an Illumina HiSeq 2500 (Illumina, San Diego, CA). On average, 43 million, 150 base pair single end reads were generated for each sample and used in the analysis. Sequencing data are available from the NCBI GEO database (experimental series accession number: GSE115188).

### 2.7. Transcript assembly, quantification, and differential expression analysis

Trimmomatic was used to remove sequencing adapters and low-quality ends [50]. The cleaned dataset was analyzed with the Tuxedo pipeline. Subsequently, readings were mapped to the mouse reference genome (Ensembl GRCm38, mm10) using TopHat2 software (http://tophat.cbcb.umd.edu/). The ‘—library_type’ parameter was set to ‘fr-firststrand’. Default settings were preserved for all other TopHat2 parameters. The resulting alignments files from TopHat2 (average mapping rate of 87.4%) were used to generate a transcriptome assembly. Gene expression was calculated for each condition using the Cufflinks (http://cufflinks.cbcb.umd.edu/) and Cuffmerge utilities. Due to the relatively small sample size of each group (*N* = 4), Cuffdiff2 with default settings was used to identify transcripts that were differentially expressed between each treatment group compared to the water only control group. This analysis strategy was chosen based on a previous research with a similar research design [51]. The significance threshold was set at *q* < 0.05 (FDR corrected) [52]. Finally, a Fisher’s exact test was performed using the GeneOverlap R package to test the significance of DEG overlaps [53].

### 2.8. Weighted Gene Co-expression Network Analysis (WGCNA)

Gene co-expression networks were identified using the Weighted Gene Co-expression Network Analysis (WGCNA) package [54]. Briefly, genes were removed if at least one value of the sixteen samples had FPKM <1. The remaining genes were log-transformed using the Log_2_(X+1) function. Following this, 12,679 genes were used to build a co-expression similarity matrix based on Pearson correlations and transformed into a signed adjacency matrix using the soft thresholding power of *β* = 18 [54]. Genes were hierarchically clustered, signed gene networks were built using minModuleSize = 20, deepSplit = 4, and similar modules were merged using mergeCutHeight = 0.1. The resulting modules were assigned to arbitrary color names. To identify modules associated with the experimental conditions, one-way ANOVAs were performed using the module eigengene (ME) value for each of the resulting modules. Correction for multiple testing was applied using false discovery rate (FDR- adjusted q-value < 0.05). Finally, Tukey’s *post hoc* test was performed to identify significant differences between experimental groups and the WW group.

### 2.9. Functional enrichment

To obtain information about possible underlying biological processes pertinent to the study, the DEG and significant WGCNA module gene lists were uploaded to Ingenuity Pathway Analysis software (IPA; Ingenuity Systems, Inc, Redwood City, California, USA, http://www.ingenuity.com). Functional enrichment for pathways restricted to mouse nervous system were performed, and scores for upstream regulators, mechanistic networks, causal networks, and downstream effects were obtained [55]. Each IPA network is scored based on the fit of significant genes in each dataset using the Fisher exact test [56].

## 3. Results

### 3.1. Behavior

#### 3.1.1. Nicotine phase

**Fig 2:**
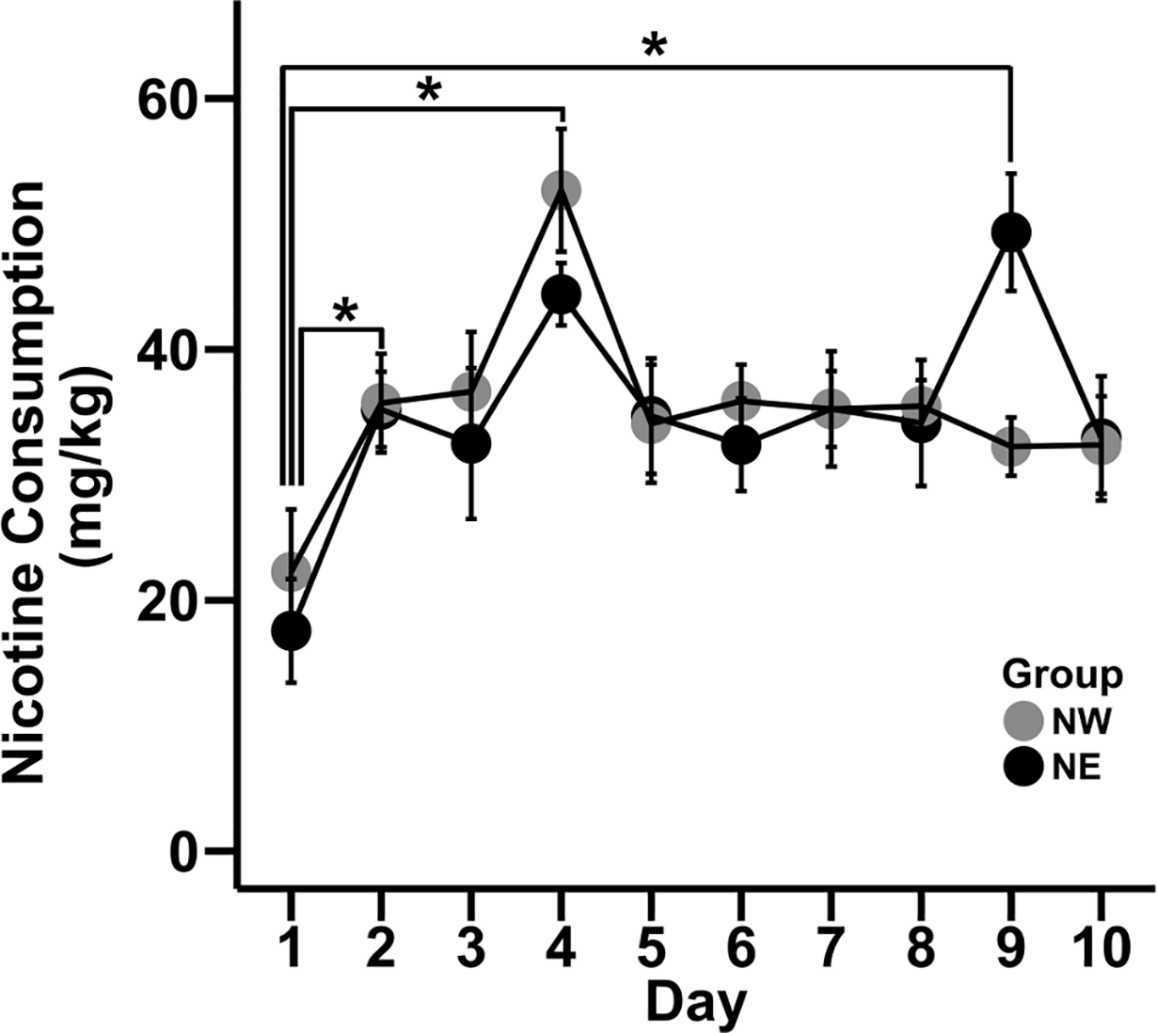
Nicotine consumption increased after the first day but remained similar between groups. Data shows 22 h nicotine consumption (mg/kg) for NW and NE mice on days 1 to 10. * represents significantly (p< 0.05) different than day 1.

Analysis of nicotine consumption (mg/kg) across study days revealed a significant main effect of day (F _9,180_ = 8.02, *p*<0.01; Fig 2). *Post hoc* analyses revealed a significant increase in nicotine consumption on days 2, 4, and 9 (all p<0.05) compared to day 1. No significant main effect or interactions with group were observed. No significant main effect of day, nicotine treatment, or day x nicotine treatment interaction were detected for the 2h water intake between days 1 to 5 in this experimental phase. Further, no significant differences in body weight were observed between groups.

#### 3.1.2. Drinking in the dark (DID)

**Fig 3:**
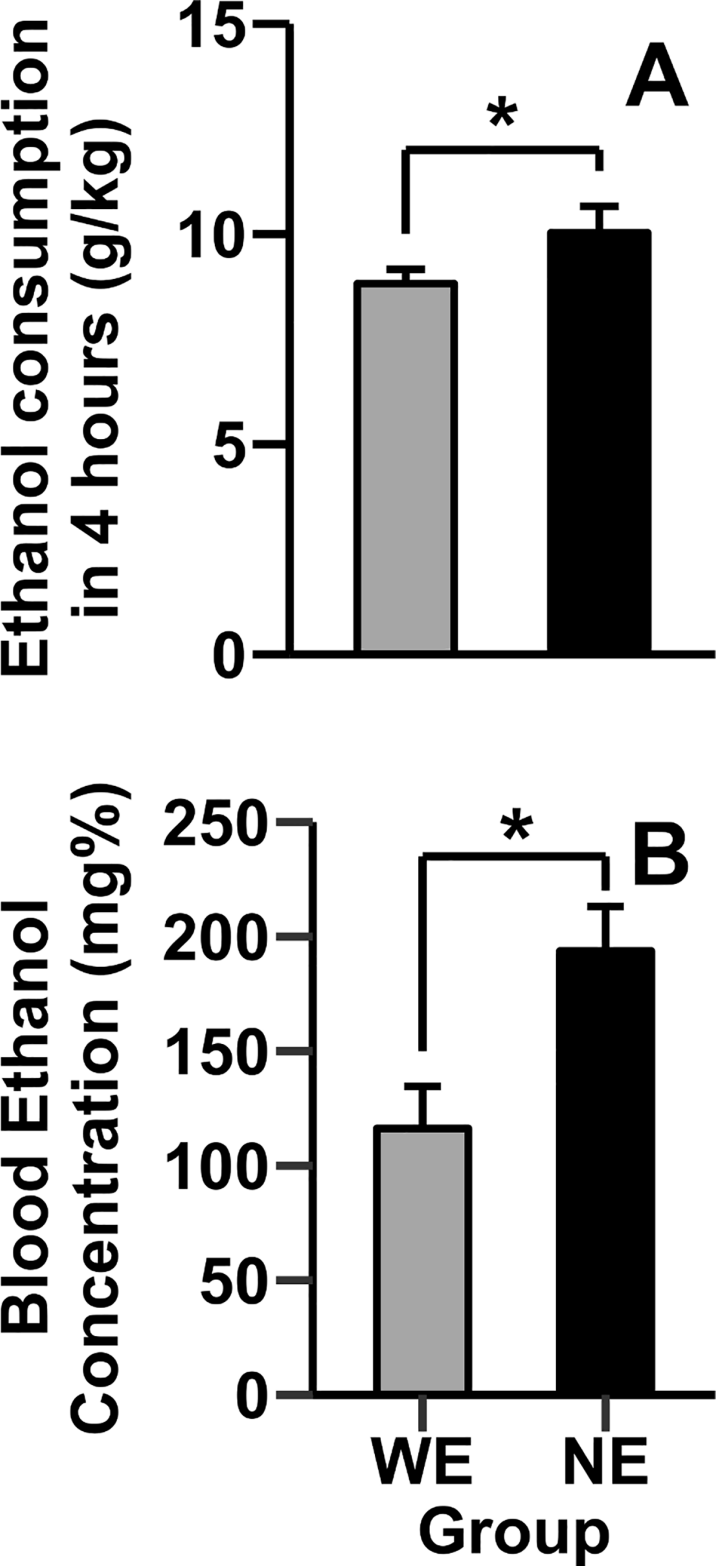
Nicotine exposure during adolescence increases ethanol consumption and resulting BEC. Data (mean ± SE) represents **(A)** 4h ethanol consumption (g/kg) on the final experimental day and **(B)** Blood Ethanol Concentration (BEC) in the WE and NE animals. * represents p<0.05.

Mice with access to nicotine during adolescence consumed significantly more ethanol and had higher BEC than mice with access to water only (Fig 3). For this analysis, we focused on the 4-hour exposure of the last experimental day, commonly used as a measure of binge-like ethanol consumption [57–61]. Replicating our prior results [35], mice exposed to nicotine consumed significantly more ethanol (*t*_14_ = 1.69, *p*<0.05; Fig 3A) and had significantly higher BEC (*t*_14_ = 2.89, *p*<0.05; Fig 3B) than nicotine naïve mice. No significant differences were found in ethanol consumption on days 7–9 (Mean ± Standard Error of Mean; Day 7: WE = 0.34 ± 0.02, NE = 0.35 ± 0.03; Day 8: WE = 0.31 ± 0.03, NE = 0.35 ± 0.05; Day 9: WE = 0.41 ± 0.06, NE = 0.42 ± 0.06). Further, no significant differences in water consumption were detected between the WW and NW groups on the last day of the experiment (1.05 ± 0.20; 1.50 ± 0.21 respectively), nor were differences in body weight between groups observed.

### 3.2. Differentially expressed genes (DEG)

**Fig 4:**
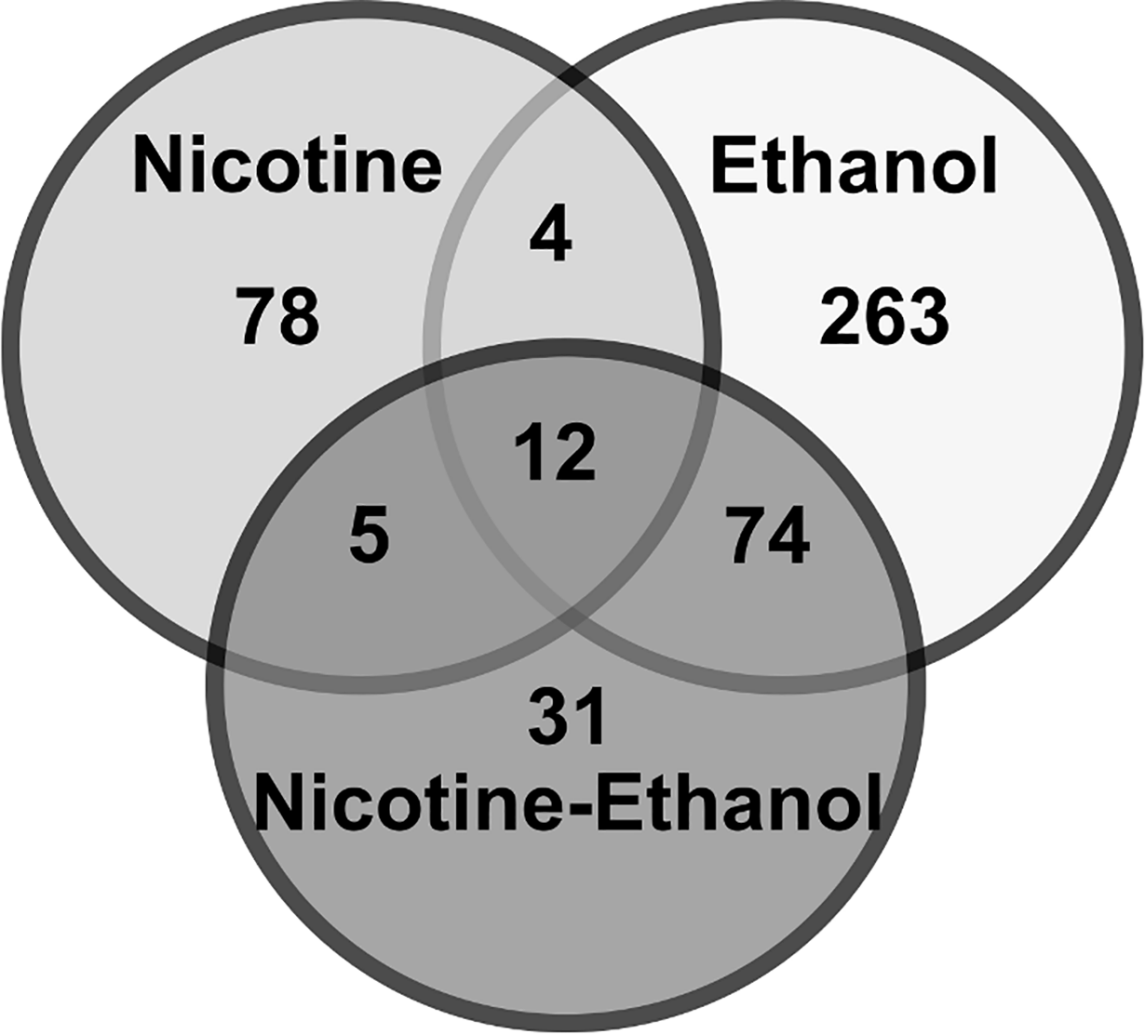
The ethanol group showed the highest number of DEG compared to the nicotine only and nicotine-ethanol groups. Venn diagram of differentially expressed genes (DEG) among three treatment groups compared to water only (FDR< 0.05) and their overlap. Sixteen DEG were shared between NW and WE groups, 17 DEG between NW and NE, and 86 DEG between WE and NE. Only 12 genes were shared among all treatment groups.

We examined DEG in each of the three drug treatments groups compared to the water only control group. Sixteen DEG were shared between the NW and WE groups, 17 DEG were shared between both groups exposed to nicotine, and 86 DEG overlapped between both groups exposed to ethanol (Fig 4). Twelve DEG were shared across all treatment groups. All overlapping DEG were significant (p<0.01).

**Fig 5:**
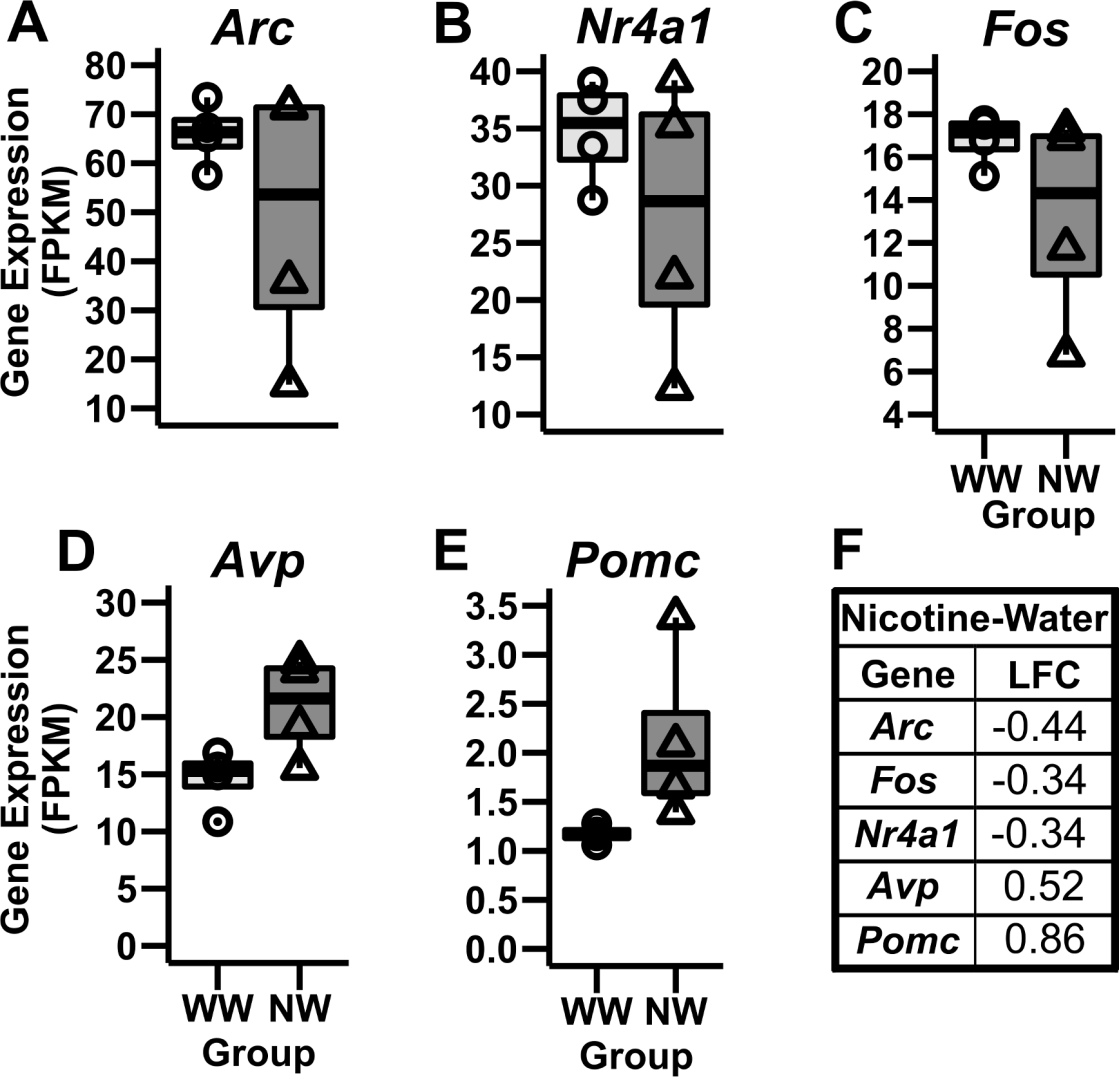
Notable genes differentially expressed between the WW and NW group. **(A- C)** The *Arc*, *Fos*, and *Nr4a1* genes showed a downregulation in the NW group compared to the WW group. **(D-E)** Inversely, *Avp* and *Pomc* genes were upregulated in the NW group. **(F)** The log-fold change (LFC) values are presented in the Nicotine-Water table.

Exposure to only nicotine (NW) resulted in 99 DEG (Fig 4 and S1 Table) at FDR < 0.05, of which 84 were downregulated and 15 were upregulated. Expression changes ranged from a logarithmic fold change (LFC) of -4.38 to 3.90 (Fig 5). Among notable genes previously associated to nicotine consumption we found an upregulation of the Pro-opiomelanocortin (*Pomc*) gene which mediates the anorectic effects of nicotine through activation of acetylcholine receptors [62,63] and the vasopressin (*Avp*) gene, involved in the facilitation of stress-induced neuronal activation and regulation of hypothalamic adrenocorticotropic hormone (ACTH) release [64]. Additionally, we observed a downregulation of the Activity Regulated Cytoskeleton-Associated Protein (*Arc*) gene, Fos Proto-Oncogene (*Fos*) gene, and Nuclear Receptor Subfamily 4 Group A Member 1 (*Nr4a1*) gene. These genes have been previously associated with neurogenesis, cell proliferation, cell differentiation, and cell transformation [65–68].

**Fig 6:**
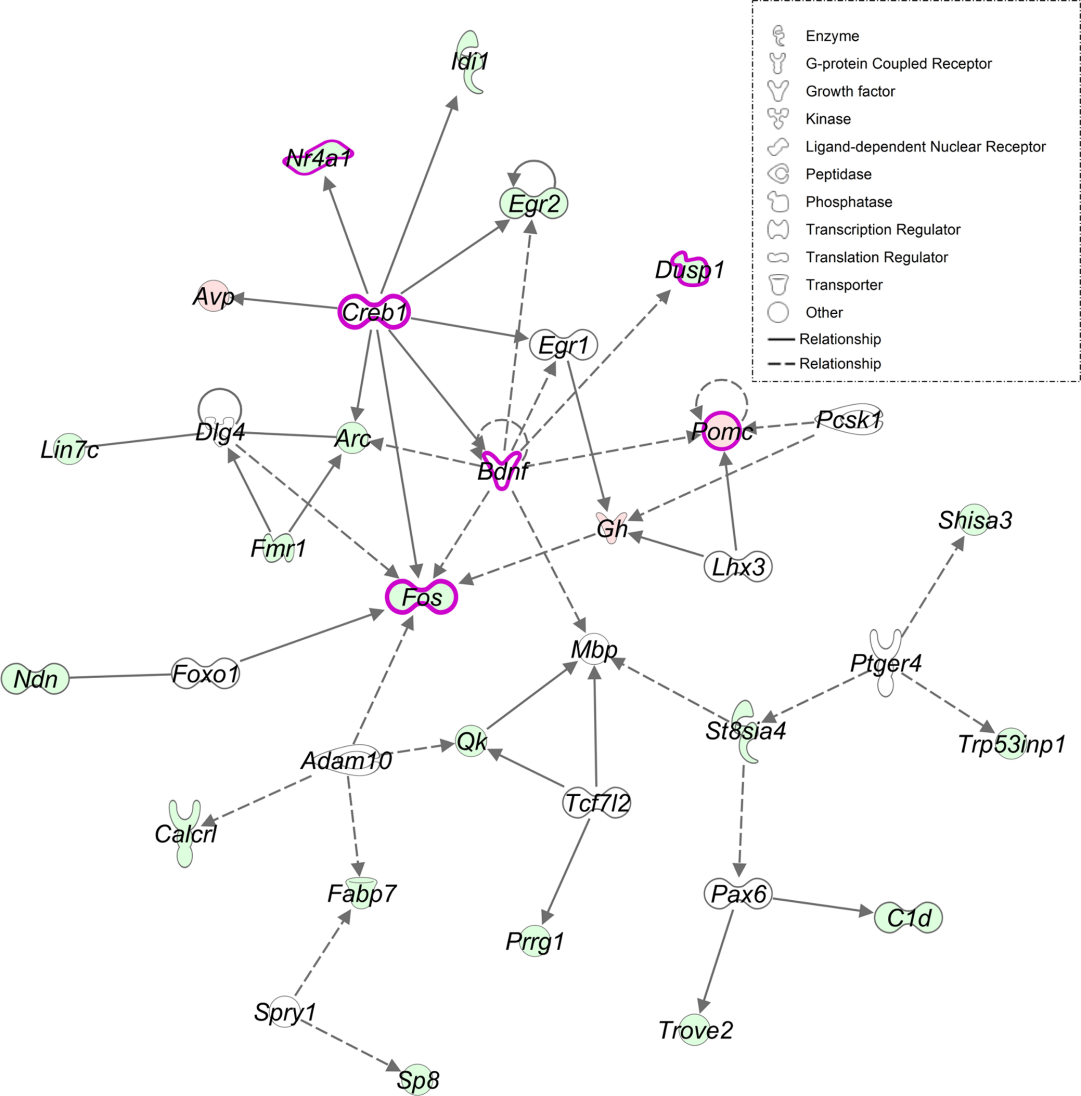
IPA network shows the associations of genes affected by nicotine exposure in adolescent C57BL/6J mice. This figure highlights genes enriched for the stress-related pathways: corticotrophin releasing hormone signaling and glucocorticoid receptor signaling (pink bold nodes corresponding to NW DEG and upstream regulators). This figure shows a direct effect of the upstream regulator gene *Creb1* on *Nr4a1*, *Bdnf*, *Fos*, *Arc*, and *Avp* genes involved in neuroplasticity and stress response. Indirect relationships are indicated for *Bdnf* on *Fos*, *Arc*, and *Pomc*. Up (light red) and down (light green) regulated genes are shown. Direct (bold arrow) and indirect (dashed arrow) relationships are displayed. IPA functional categories are shown in node key.

Using the NW DEG, IPA identified an enrichment of the corticotrophin releasing hormone signaling pathway (4 genes, p<0.05: Fig 6). Relevant upstream regulators included the Corticotropin-Releasing Hormone Receptor 1 (*Crhr1*), CAMP Responsive Element Binding Protein 1 (*Creb1)*, Brain-Derived Neurotrophic Factor (*Bdnf*), and dopamine release such as Dopamine Receptor D2 (*Drd2*) (Table 1). These genes have been associated to nicotine exposure in prior studies [69–73].

**Table 1:** Upstream regulators of NW DEG identified by IPA.

DEG Nicotine-Water: IPA functional over-representation		
Upstream Regulators	p-value of overlap	Target molecules
***Creb1***	8.23E-08	***↑*** *Avp*, ***↓*** *Arc*, ***↓*** *Fos*, ***↓*** *Egr2*
***Drd2***	1.15E-04	***↑*** *Cshl1*, ***↑*** *Pomc*, ***↓*** *Fos*
***Bdnf***	4.05E-04	***↑*** *Pomc*, ***↓*** *Fos*, ***↓*** *Arc*, ***↓*** *Cav2*, ***↓*** *Egr2*, ***↓*** *Nr4a1*
***Crhr1***	6.17E-03	***↑*** *Avp*

This table contains the upstream regulators identified using IPA and their target molecules, corresponding to genes present in the list of DEG following nicotine treatment (↑ = upregulated; ↓ = downregulated).

**Fig 7:**
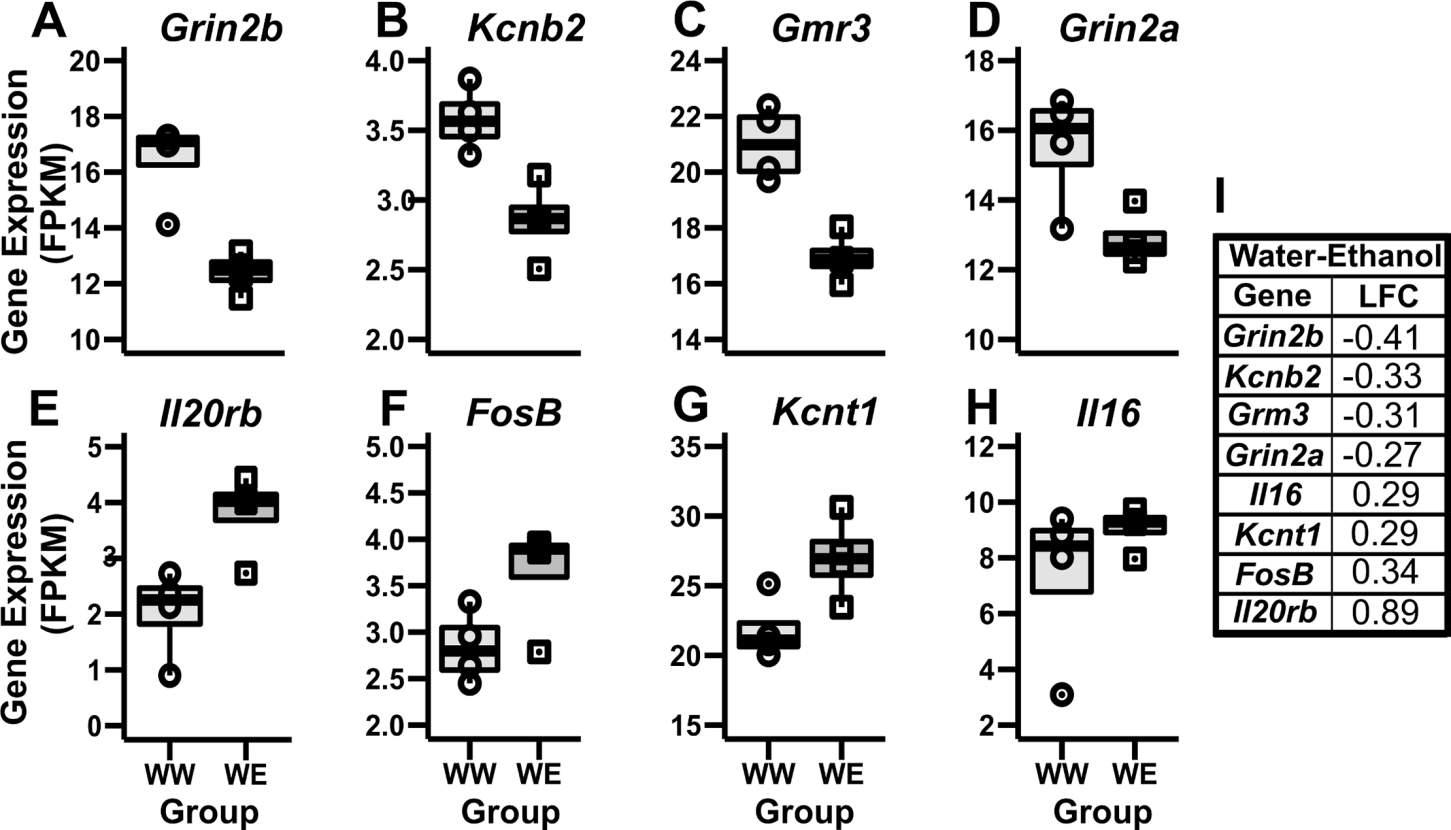
Notable genes differentially expressed between the WW and WE groups. **(A-D)** The *Grin2b*, *Kcnb2*, *Grm3* and *Grin2a* genes were downregulated in the WE group compared to the WW group. **(E-H)** *Il16*, *Kcnt1*, *FosB* and *Il20rb* genes were upregulated in the WE group. **(I)** The log-fold change (LFC) values are presented in the Water-Ethanol table.

Exposure to only ethanol (WE) resulted in 353 DEG (Fig 4 and S2 Table) of which 268 were downregulated and 85 were upregulated with an LFC ranging from -1.68 to 4.61. Notable DEG identified for the ethanol only group were associated with neurogenesis (*FosB*), voltage-gated ion channels (*Kcnt1* and *Kcnb2)*, immune system (*Il16* and *Il20rb)*, and glutamatergic neurotransmission (*Grin2a*, *Grin2b* and *Grm3*). We observed increased expression of the FBJ Murine Osteosarcoma Viral Oncogene Homolog B (*FosB*), the Potassium Sodium-Activated Channel Subfamily T Member 1 (*Kcnt1)*, the Interleukin 16 (*Il16)*, and Interleukin-20 receptor subunit beta precursor (*Il20rb*) genes, implicated in immune responses and cytokine signaling (Fig 7A–7D). There was decreased expression of the Subfamily B Member 2 (*Kcnb2*), Glutamate Metabotropic Receptor 3 (*Grm3)*, Glutamate Ionotropic Receptor NMDA-Type Subunit 2A (*Grin2a*), and Subunit B (*Grin2b*) genes (Fig 7E–7H). These genes have been previously reported to be altered by ethanol exposure and involved in ethanol sensitivity [74–80].

The WE DEG were enriched for pathways involved in hepatic stellate cell activation (10 genes, p<0.05) and intrinsic prothrombin activation (3 genes, p<0.05). Relevant upstream regulators for the WE group were Glutamate Metabotropic Receptor 2 (*Grm2)*, *Grin2a*, Serum/Glucocorticoid-Regulated Kinase (*Sgk1*), Adenylate Cyclase Activating Polypeptide 1 (*Adcyap1)*, Adenylate Cyclase 5 (*Adcy5)*, and *Bdnf* (Table 2). These genes have been previously reported to be altered by ethanol [81–84].

**Table 2:** Upstream regulators of WE DEG identified by IPA.

DEG Water- Ethanol: IPA functional over-representation		
Upstream Regulators	Overlap p-value	Target molecules
***Gmr2***	1.22E-03	***↓*** *Grin2a*, ***↓*** *Grm3*
***Adcyap1***	2.03E-03	***↑*** *Col5a1*
***Adcy5***	3.96E03	***↑*** *Adcy6*, ***↑*** *FosB*
***Sgk1***	2.39E-03	***↓*** *Grin2a*, ***↓*** *Grin2b*
***Bdnf***	2.70E-02	***↓*** *Grin2a*

This table contains the upstream regulators identified by IPA and their target molecules, corresponding to genes differentially expressed following ethanol administration (↑ = upregulated; ↓ = downregulated).

**Fig 8:**
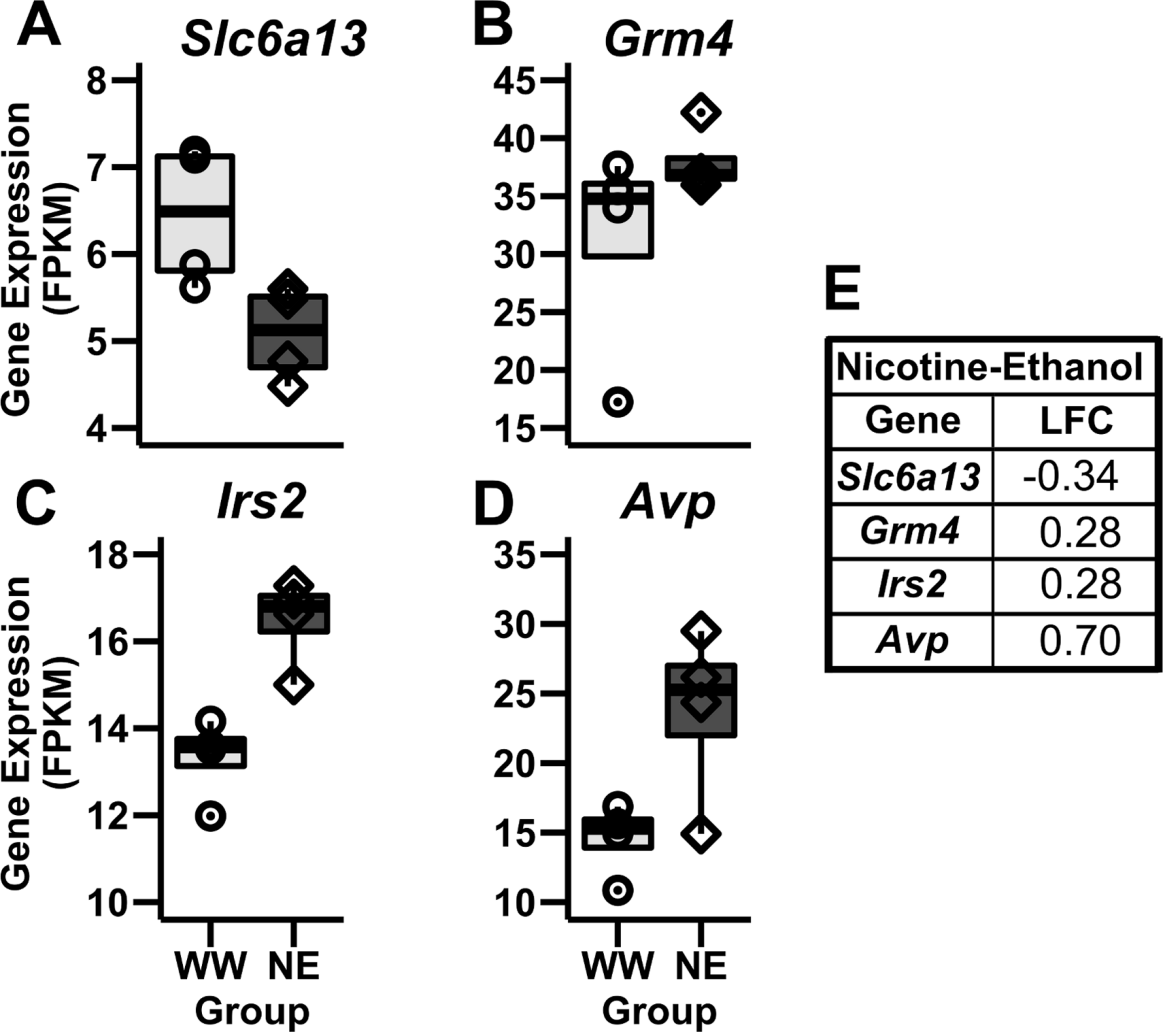
Notable genes differentially expressed between the WW and NE groups. **(A-B)** The *Slc6a13* gene was downregulated in the NE group compared to the WW group. **(C-D)** *Grm4*, *Irs2* and *Avp* genes were upregulated in the NE group. **(E)** The log-fold change (LFC) values are presented in the Nicotine-Ethanol table.

Nicotine and ethanol exposure (NE) resulted in 122 DEG (Fig 4 and S3 Table), of which 46 genes were downregulated and 76 upregulated, with an LFC range of -2.44 to 1.40. There was an upregulation of relevant DEG previously implicated in both nicotine and ethanol consumption such as *Avp* [85–87] and the Metabotropic glutamate receptor 4 (*Grm4*) gene [33,74]. Additionally, the Solute Carrier Family 6 (Neurotransmitter Transporter, GABA) Member 13 (*Slc6a13*) gene was downregulated in this group (Fig 8).

IPA of DEG from the NE group identified enrichment of dendritic cell maturation (6 genes, p<0.05) and synaptic long-term depression (4 genes, p<0.05) pathways. The upstream regulator genes had similar function to those identified for our nicotine only and ethanol only groups. These genes were associated with HPA-axis function such as *Crhr1* and Corticotrophin Releasing Hormone Binding Protein *(Crhbp*). Moreover, they were implicated in transcription regulation including Transcription Factor 7 Like 2 (*Tcf7l2)*, Neurogenic Locus Notch Homolog Protein 3 (*Notch3)*, and *Notch1*. Finally, we observed upstream regulator genes associated with immune response such as Interleukin 10 (*Il10*) and oxidative deamination of dopamine, norepinephrine, and serotonin such as Monoamine Oxidase A (*Maoa*) gene (Table 3).

**Table 3:** Upstream regulators of NE DEG identified by IPA.

DEG Nicotine- Ethanol: IPA functional over-representation		
Upstream Regulators	Overlap p-value	Target molecules
***Notch3***	7.76E-03	***↓*** *Fabp7*
***Notch1***	7.76E-03	***↓*** *Fabp7*
***Crhr1***	7.76E-03	***↑*** *Avp*
***Crhbp***	1.55E-02	***↑*** *Avp*
***Tcf7l2***	2.49E-02	***↓*** *Mal*
***Maoa***	4.57E-02	***↑*** *Avp*
***Il10***	4.57E-02	***↓*** *Mrc1*

This table contains the upstream regulators identified by IPA and their target molecules, corresponding to genes differentially expressed after NE treatment (↑ = upregulated; ↓ = downregulated).

### 3.3. Weighted Gene Co-expressed Network Analysis (WGCNA)

All sixteen samples produced 140 modules in a single WGCNA analysis. The gray module contained 263 unassigned genes. The remaining 139 modules contained between 20 and 957 genes. To identify relevant modules, the ME was calculated for each experimental group. A one-way ANOVA with multiple testing correction (*q*<0.05) identified 15 modules significantly different between experimental conditions (S5 Table), containing between 23 and 741 genes (S6 Table). Tukey’s *post hoc* analysis revealed significant differences between specific groups for each module (S7 Table), with the majority of significant modules significantly different when comparing WE to WW.

Functional overrepresentation analysis using IPA found enrichment in our 15 significant modules (detailed information in S8–S22 Tables). Enriched functional pathways were associated with synaptic signaling (blue, palevioletred3, lightcyan, paleturquoise, darkred, steelblue, powderblue, darkgray, salmon and black module), immune response (lighcyan module), transcription and methylation (powederblue, black and antiquewhite1 module), amino acid biosynthesis (brown4 and antiquewhite4 module), prothrombin activation pathways (salmon module), and cell cycle regulation (salmon and black module).

**Fig 9:**
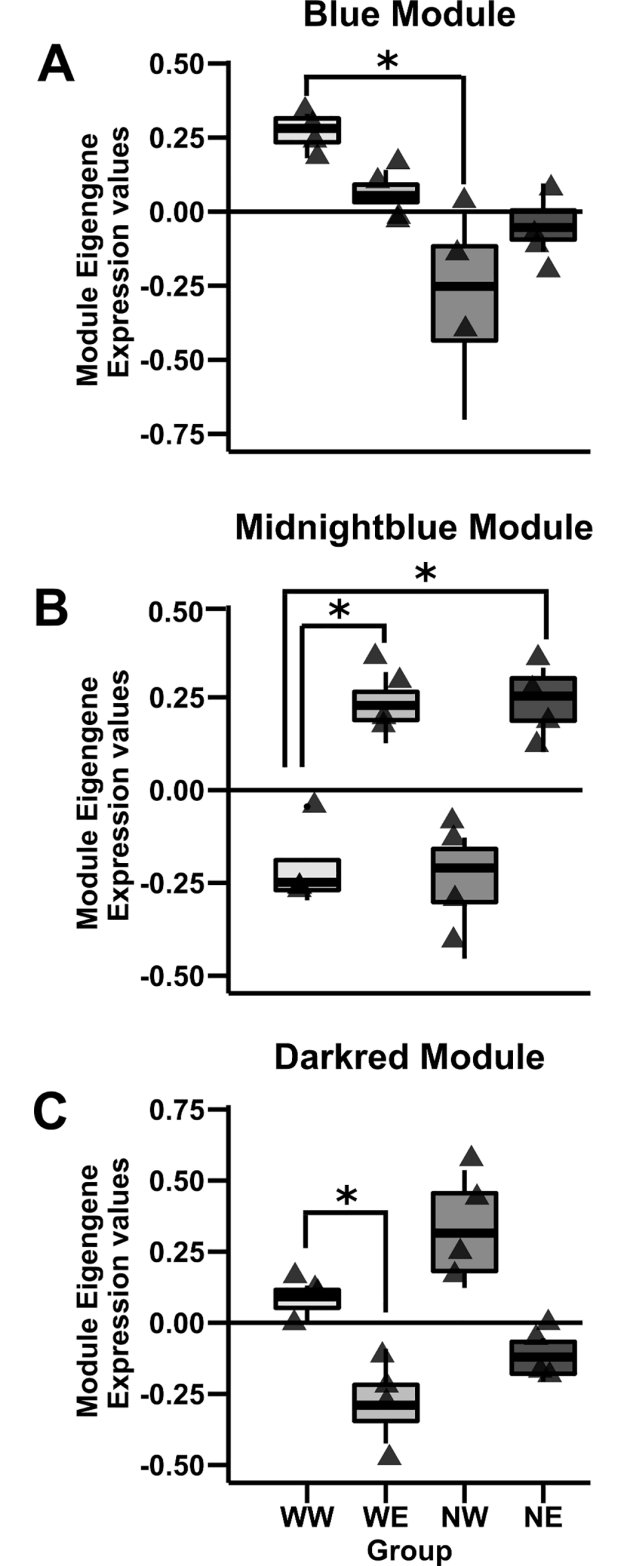
Nicotine, ethanol and their combination alter module eigengene expression values compared to controls (WW). **(A)** The Blue module shows a significant decrease in module eigengene expression in the NW group compared to the WW group. **(B)** The Midnightblue module showed a significant increase in the module eigengene for the ethanol groups (WE and NE) compared to our control group. **(C)** The Darkred module showed a significant decrease in WE module eigengene value compare to the WW group. * = p<0.05.

**Fig 10:**
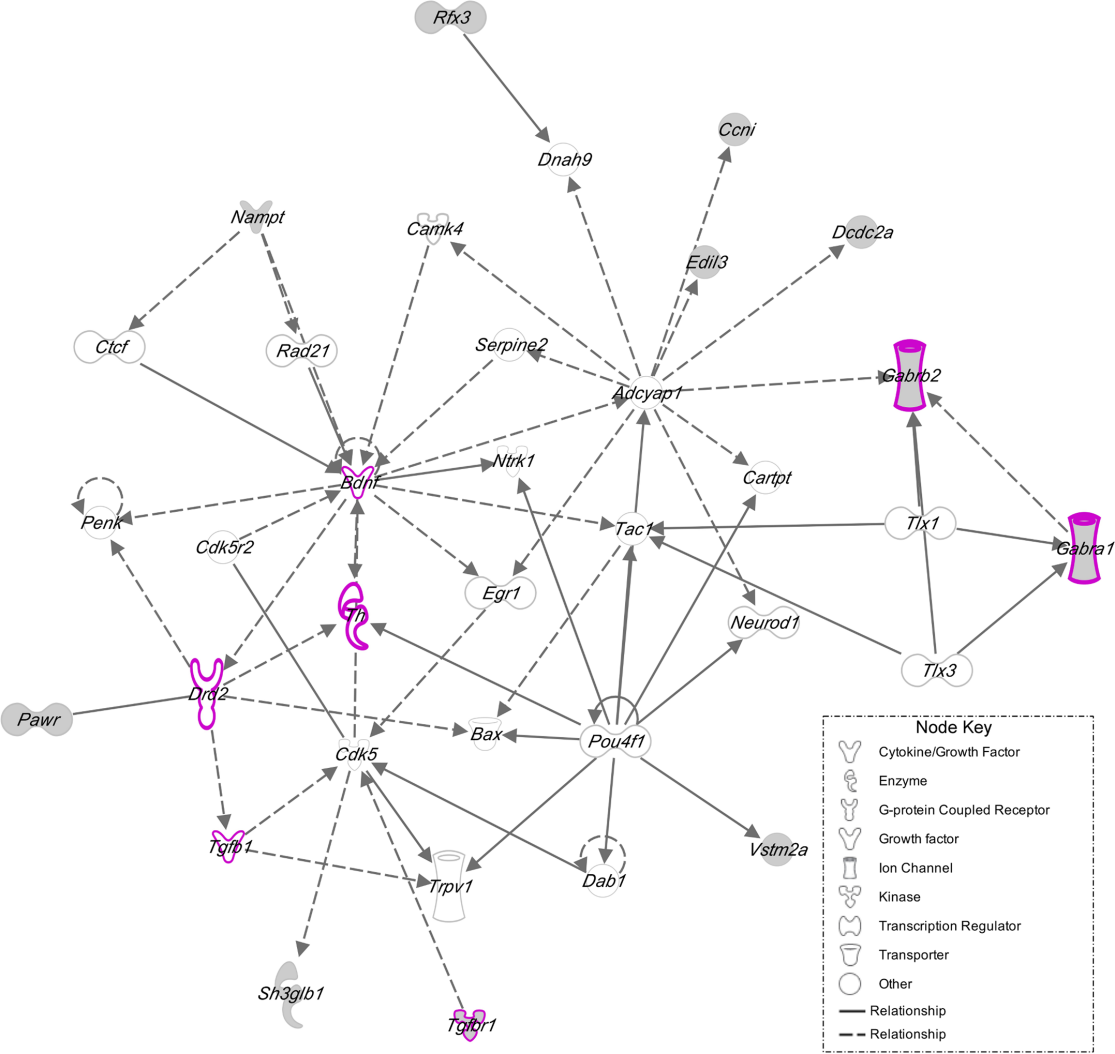
IPA enrichment of Blue module network shows the relationships of genes involved in neuroinflammation, dopamine receptor signaling, and corticotrophin hormone release pathways. This figure highlights genes (pink bold nodes) enriched for neuroinflammation signaling pathway (*Bdnf*, *Gabra1*, *Gabra2*, *Tgfb1* and *Tgfbr1*), dopamine receptor signaling (*Drd2* and *Th*), and corticotrophin releasing hormone pathway (*Bdnf* and *Campk4*). This figure shows as main nodes the *Bdnf* and *Adcyap1* (PACAP) genes involved in neurogenesis and mediators of neuroendocrine stress responses, respectively. Indirect relationships of *Bdfn* are indicated for *Campk4*, *Th* and *Drd2*. *Adcyap1* shows indirect association with *Gabra1* on *Gabra2* genes. Direct (bold arrow) and indirect (dashed arrow) relationships are displayed. IPA functional categories are shown in node key.

There were three modules (Blue, Midnightblue and Darkred) containing genes previously associated with addiction and brain development that are described below. The Blue module, containing 741 genes was significantly different between groups (*F*_(3,12)_ = 8.16, *q*<0.05). The *post hoc* analysis revealed that the NW group had less expression compared to the WW group (Fig 9A). This module contained genes such as Gamma-Aminobutyric Acid Type A Receptor Subunit Beta 2 and Alpha 1 (*Gabrb2* and *Gabra1)*, Serotonin 5-HT-2C Receptor (*Htr2c*), Voltage-Sensitive Potassium Channel (*Kcnd2*) and Insulin-Like Growth Factor 1 (*Igf1*). GABA signaling-related genes have been identified as susceptibility *loci* and genes for nicotine dependence and alcoholism [88]. Functional enrichment using IPA, revealed Ephrin receptor signaling (*p* = 0.0003), ERK/MAPK signaling (*p* = 0.001), TGF-β Signaling (*p* = 0.002) and, BMP signaling pathways (*p* = 0.002). The top network was enriched in genes associated with pathways involved in neuroinflammation, dopamine receptor signaling, and corticotrophin releasing hormone (Fig 10).

The Midnightblue module containing 168 genes, was significantly different between groups (*F*_(3,12)_ = 22.53, *q*<0.05). Tukey’s *post hoc* test revealed an increase in gene expression in both of the ethanol groups (WE and NE) compared to our control group (WW) (Fig 9B). This module contained genes such FBJ Murine Osteosarcoma Viral Oncogene Homolog (*Fosb)* previously shown to be induced by ethanol exposure [80]. Sodium/potassium/calcium exchanger (*Slc24a4*) and Cysteine-Rich Angiogenic Inducer 61 (*Cyr61*) involved in cholinergic regulation of synaptic plasticity [89] were observed here. Furthermore, Adrenoceptor Alpha 1A (*Adra1a*) previously reported in animal models of addiction and considered to be directly involved in substance use and dependence [90, 91] was found in this module. Finally, we observed the Autism Susceptibility Candidate 2 (*Auts2*) gene reported to be associated to alcohol sensitivity [91]. IPA enrichment of functional groups for the Midnightblue module indicated apoptosis receptor signaling (*p* = 0.003) pathway.

Finally, the Darkred module containing 119 genes was significantly different between groups (*F*_(3,12)_ = 16.22, *q*<0.05). *Post hoc* analysis revealed a decrease in WE module eigengene value compare to the WW group. Relevant genes contained in this module were the Glutamate Metabotropic Receptor 2 (*Grm2*) previously reported as a candidate gene in nicotine and ethanol exposure [92], RNA Polymerase I Subunit E (*Polr1e*), and G Protein Subunit Alpha O1 (*Gnao1*), which have both been shown to be altered by psychostimulants [93] (Fig 9C).

## 4. Discussion

The current study found that adolescent nicotine exposure alters later drug behavior and the expression of genes involved in glutamate and GABA neurotransmission, neuroplasticity, and the HPA-axis stress response. Our results replicate and extend previous findings, showing that nicotine exposure in adolescent C57BL/6J female mice increases binge-like ethanol consumption and resulting BEC compared to nicotine-naïve mice [35]. Furthermore, our transcriptome analyses suggest that nicotine and ethanol exposure results in alterations of brain neuroendocrine- (e.g. *Avp*, *Pomc*, and *Crhr1*), neuroplasticity- (e.g. *Arc*, *Fos*, *FosB*, *Nr4a1* and *Bdnf*), and neurotransmitter-related (e.g. *Drd2*, *Grin2a*, *Grin2b*, *Grm3*, *Grm4*, and *Kcnt1*) genes. These findings contribute additional evidence on the role of nicotine-induced changes in stress hormone signaling and nicotine-induced neuroadaptations that may lead to increased ethanol self-administration [32]. Moreover, our WGCNA analysis allowed us to contextualize the effects of early nicotine or/and ethanol consumption on biological pathways associated to brain development and function. This research provides information about possible underlying transcriptional changes and biological mechanisms associated with nicotine and/or ethanol consumption during adolescence, that should be further examined.

We found that nicotine exposure increases binge-like ethanol consumption and BEC in female adolescent C57BL/6J mice. Further, no significant differences in water consumption were detected between the NW and WW groups, indicating that nicotine exposure does not globally increase thirst. Our results are consistent with previous studies that have reported a significant increase in ethanol consumption after nicotine exposure in adolescent C57BL/6J male [94,95] and female [35] mice, adult C57BL/6J mice [94], male rats [24,30–32,96–98], and human smokers [99]. While two studies did not find a relationship between nicotine consumption and ethanol intake [100,101], the preponderance of evidence suggests that this relationship exists. Our data provides further evidence for this association. One mechanism proposed to explain the increase in ethanol intake following nicotine exposure is stress-hormone signaling in the mesolimbic dopamine (DA) system. Corticosterone in response to nicotine has been shown to increase GABAergic inhibition onto VTA DA neurons leading to attenuated ethanol-induced DA signaling and augmented ethanol self-administration [32]. Gene expression results observed in this study are consistent with this hypothesis (see Conclusions section).

### Genes influenced by nicotine exposure

Among humans, nicotine use usually begins during adolescence [102]. This developmental period represents a window of time where normal brain development occurs, which may be altered by nicotine exposure. Alterations to normal neuronal development can increase the risk of future drug use [32,87,103]. During this time, many systems undergo changes, including the mesocorticolimbic DA system [104] and the hypothalamic-pituitary-adrenal (HPA) axis [105].

Perturbations of HPA axis developmental trajectories may contribute to altered stress-reactivity [105]. Nicotine activates the HPA system which stimulates stress-related hormones [106], that can modulate synaptic transmission in the mesolimbic DA system [37]. Both the DA and HPA-axis have been linked to drug use and addiction [107]. Here we showed that nicotine exposure in C57BL/6J adolescent females increased the expression of genes involved in neurotransmission (*Pomc*) and in neuropeptide activity (*Avp*) relative to animals that had access to water; both genes linked to HPA axis activity. Further, *Crhr1* gene was identified as upstream regulator of the NW DEG.

Our results are consistent with previous studies that have identified increased expression of these genes following nicotine exposure [87,108–110], but opposite results have also been reported [111,112]. For example, *Pomc* upregulation has been observed in the arcuate nucleus of the hypothalamus after chronic nicotine treatment [108,110]. However, a decrease or no effect in *Pomc* mRNA expression has been observed in the same brain region after chronic nicotine treatment [112,113] and acute nicotine treatment [111,112]. These discrepant findings may be explained by differences in the nicotine administration protocol. For example, in our experiment 200 μg/ml (−)nicotine was available in the drinking solution for ten days, compared to one-time [111] or daily [112] injections of nicotine utilized in the experiments reporting decreased *Pomc* expression. Further, we choose to examine whole brain gene expression, therefore it is possible that brain region-specific expression changes for the *Pomc* gene [114] were washed out. Finally, in this work we analyzed the brain transcriptome of C57BL/6J adolescent female mice; therefore, our results might not be comparable due to differences in species, sex or age of animal tested. In contrast to the mixed results with *Pomc*, *Avp* was upregulated in our nicotine treated animals, a finding that is consistent with previous literature [69,87,109]. Together these data suggest that nicotine alters HPA-axis activity through increases in *Avp* and *Pomc* gene expression, however the later seems to be susceptible to treatment protocol and/or brain region examined.

In addition to our results which demonstrate that nicotine alters genes associated with HPA axis activity, we also found that nicotine alters GABAergic transmission. Further, it has been reported that stress signaling alters GABAergic neurotransmission [103]. This is supported by the identification of the stress-related gene *Crhr1* gene as upstream regulator and a decreased expression of relevant genes in the Blue module (*Gabrb2* and *Gabra1)* in the NW group. Therefore, our findings contribute additional evidence to the hypothesis that nicotine utilizes neuroendocrine mechanisms to influence neurotransmitter activity [32,69].

We observed decreased *Arc* and *Fos* expression in the NW group relative to controls. These genes have been implicated in synaptic plasticity [115] and associated with addiction [113]. Our results are not consistent with previous research that has reported increased *Arc* and *Fos* expression in the prefrontal cortex (PFC) following an acute injection of nicotine in adolescent male rats [113] and rat pups [116]. It is possible that we observed a different pattern of gene expression due to the brain region studied. Particularly, *Arc* and *Fos* brain region expression depends on region-specific neuronal activation [117]. On the other hand, these gene expression patterns could be explained due to treatment protocol (i.e. chronic), or as a result of the 4h nicotine withdrawal on the final experimental day.

A possible explanatory mechanism to our results is that AMPA glutamate receptors activation have shown to negatively regulate *Arc* gene expression, through a mechanism involving a pertussis toxin-sensitive G proteins [118]. Interestingly, nicotine stabilizes ionotropic glutamate receptors leading to increased AMPA function in rats [119,120]. Thus, we could hypothesize that decreased *Arc* expression could be a result of nicotine increasing AMPA receptor function and resulting in decreased *Arc* expression. This negative feedback mechanism may only be apparent with chronic nicotine exposure. Further work measuring *Arc* gene expression after acute and chronic nicotine exposure is required to test this hypothesis.

### Genes influenced by ethanol exposure

Mice that consumed only ethanol had more DEG compared to any other treatment group. The DEG found in this group were generally consistent with previous research that examined genes related to ethanol consumption and/or BEC in C57BL/6J mice utilizing DID. Importantly this consistency comes with many differences in study design. For example, we used female mice compared to male mice in previous work. Additionally, we used whole-brain samples while prior work focused on specific brain regions such as the hippocampus, striatum, cerebellum, frontal cortex [121,122], olfactory bulb, and VTA [123]. Even with these methodological differences overlap in DEG were observed and these genes (e.g. *Cav2*, *Hbb-b1*, *Col6a1* and *Col7a1*) could represent key drivers of ethanol intake.

Among our notable results, the transcriptome results showed decreased expression of glutamate receptor genes such as *Grm3* (encoding the metabotropic receptor subunit mGluR3), *Grin2a*, and *Grin2b* (NMDA type, encoding for GluN2A and GluN2B respectively) compared to controls. Glutamate is responsible for normal brain function during development [124] and has an important role in synaptic plasticity and excitatory synaptic neurotransmission [74]. Long-term exposure to ethanol alters the gene expression, the availability and function of glutamate receptors, and its transporters [125]. Further, impairment in glutamate homeostasis has been associated with alcohol tolerance, dependence, and relapse [75]. Our results are consistent with previous studies in human embryonic stem cells (hESCs) [75] and human post mortem brain tissue of chronic alcoholics [126]. However, opposite results have been reported as well. Particularly, the upregulation of the NMDA receptor subunit genes *GRIN2A* and *GRIN2B* have been reported in the hippocampus of human alcoholics [74], in hESC-derived cortical neurons [75], in mouse cortex [127], and in rats cortex [128] and amygdala [129] after long-term ethanol intake. It has been proposed that acute ethanol exposure reduces glutamatergic transmission, while prolonged exposure upregulates NMDA receptor function [130] and transcription [131,132]. Therefore, it is possible that a 4-day DID treatment was not long enough to observe the switch between reduced transmission and upregulation of NMDA receptor expression.

We observed that ethanol consumption induced *FosB* transcription in our experiment. This gene was found in both the WE DEG and Midnightblue module. *FosB* has been associated with addiction-related neural plasticity [133]. Long-lasting induction of FosB is related to chronic stress [134], drug abuse [78] as well as, ethanol exposure [80,135]. Our results are consistent with previous studies in animal models after chronic voluntary ethanol intake, showing an upregulation of *FosB* gene expression [80]. However, opposite results have been reported regarding *FosB* differential expression in the striatum and mPFC after forced ethanol exposure [80,134]. This could suggest that *FosB* is sensitive to ethanol exposure protocols.

Finally, our WGCNA results revealed that the majority of the significant modules were related to ethanol exposure (S7 Table). A few of these ethanol-responsive modules had axonal guidance signaling functionally overrepresented. Disruption of axon outgrowth has been reported in the developing hippocampus. These changes have been associated altered functional properties of synaptic circuitry, linked to cognitive and behavioral problems [136].

### Genes influenced by both nicotine and ethanol exposure

Mice that consumed both nicotine and ethanol showed an upregulation of the stress-related gene *Avp*. Nicotine stimulates the HPA-axis by inducing the co-expression of *Crf* and *Avp* [87]. The activation of the stress pathways is mediated by the CHR-R1, CHR-R2 and AVP V1b receptors [69], located in the amygdala, hypothalamus, anterior pituitary, and hippocampus [137]. Additionally, AVP has been implicated in ethanol drinking [85] and the AVP V1b receptor has been shown to modulate ethanol self-administration [137]. Our results are consistent with our DEG results in the NW group and with previous studies reporting an upregulation of *Avp* mRNA after nicotine exposure [87]. Conversely, no effect of acute ethanol injection [138] or decreased *Avp* mRNA levels after chronic ethanol exposure in rats (6 or 10 months) [139], provide evidence of long-term effects of ethanol on *Avp* gene expression. Therefore, we hypothesize that nicotine induced *Avp* gene expression as 4 days of ethanol exposure might not have been long enough to cause decreased *Avp* mRNA as reported in previous studies.

We observed decreased expression of the GABA transporter gene *Slc6a13* (encoding for GAT2) after both nicotine and ethanol exposure. A downregulation of other GABA-related genes (*Gabra1* and *Gabrb2*) was also observed in our WGCNA results (in the Blue module). Although there is not much evidence regarding the functional importance of GAT2 in the brain, it has been suggested to regulate cerebrospinal fluid GABA concentration [140]. A previous study reported an association between *Slc6a13* upregulation in the striatum and ethanol consumption [141]. Our results are not consistent with this study, however we observed decrease in *Slc6a13* mRNA following both nicotine and ethanol exposure. Further research regarding the function of *Slc6a13* in substance use is needed.

The WE group had the highest number of DEG (*N* = 353) compared to the NW (*N* = 99) and NE (*N* = 122) groups. It has been proposed that nicotine exposure blunts ethanol-induced synaptic function and excitatory neuron firing through stress hormone signaling [32]. We hypothesize that the decreased neuronal firing in response to nicotine exposure may also block induction of gene expression by ethanol. In such the animals exposed to nicotine would have less of a physiological response to ethanol, which would explain the significant increase of ethanol consumption observed in the NE compared to the WE group. On the other hand, it is possible that differences in the number of DEG between the NE and WE groups could be a result of ethanol consumption. However, it is important to highlight that the NE group consumed more ethanol than the WE group, but had fewer DEG.

In the current study, we also found interesting novel genes. For example, an upregulation of interleukin-like genes (*Il16* and *Il20rb*) and potassium channel genes (*Kcnt1* and *Kcnb2*, up and downregulated respectively) in the ethanol-only group. These genes could be interesting new candidate genes because of the suggested role of neuroinflammation in ethanol consumption [77]. Additionally it has been reported that potassium channels are direct targets of ethanol [76,77] and a GWAS study has found an association between a *Kcnb2* single nucleotide polymorphism (SNP) and maximum number of drinks in a human population study [76].

### Limitations

The limitations of this study were: (1) the use of whole brain tissue for RNA-Seq. This did not allow us to associate specific transcriptional changes to specific brain regions or cell types. Therefore, the inferences regarding gene expression changes and associations with behaviors are limited. Additionally, differences or the absence of DEG previously reported as highly associated with nicotine and ethanol consumption (e.g. nAChRs, glucocorticoid receptors, etc.) might due to complex gene expression patterns (up or downregulation) in different brain regions. However, it is important to highlight that we have been able to observe relevant DEG in whole brain tissue demonstrating the importance and extending the relevance of these genes across brain regions. (2) Only female adolescent C57BL/6J mice were used, due to their reported increased susceptibility to binge drinking during adolescence. However, future studies comparing C57BL/6J mice with ethanol avoiding strains could yield valuable insight into the molecular changes associated with susceptibility to drinking. (3) Ethanol exposure occurred during the 4 hours prior to brain dissection while no nicotine was present. This could explain why we observed less DEG in the NW group compared to the WE and NE groups and why the majority of the WGCNA modules were related to ethanol exposure. Further studies could include a nicotine minipump during ethanol exposure to test if the differences in the number of DEG or WGCNA modules between groups are related to time-dependent effects or more interestingly, corresponds to different actions of each of these substances. Finally, it is possible that effects observed in the nicotine group represent genes associated with withdrawal from nicotine rather than effects of nicotine intake per se. (4) While we randomly selected the mice for the RNA-Seq analysis, the small sample size in each group (*N* = 4), could be prone to sampling bias. Previous work has shown robust results with a similar samples sizes [142,143]. However, for WGCNA, 16 samples are just above the recommended sample size and could have resulted in noise in the network construction. Further study and validation of these results is warranted.

## 5. Conclusions

This study is one of the first to describe the effects of adolescent nicotine exposure on ethanol intake and the combined effect of both substances on brain gene expression. Here we observed that nicotine exposure increases ethanol consumption and resulting BEC in female adolescent C57BL/6J mice. Based on our results, we hypothesize that nicotine-induced upregulation of stress-related genes (*Crhr1*, *Avp* and *Pomc*) could be affecting GABAergic, DAergic, and glutamatergic neurotransmission in the mesolimbic pathway. This would increase glutamatergic activity and would reduce the inhibitory control of GABA on DAergic transmission [32], resulting in increased ethanol consumption observed in the NE group. Nonetheless, mechanistic experiments are required to test this hypothesis. Given the limitations of this study, validation of these findings is warranted.

## Supporting information

S1 Table List of differentially expressed genes (DEG) between Nicotine-Water and Water-Water group. (XLSX)

S2 Table List of differentially expressed genes (DEG) between Water-Water and Water-Ethanol group. (XLSX)

S3 Table List of differentially expressed genes (DEG) between Nicotine-Ethanol and Water-Water group. (XLSX)

S4 Table Overlap of differentially expressed genes (DEG) between experimental groups. (XLSX)

S5 Table ANOVA of the MEs (adjusted p-values using FDR). (XLSX)

S6 Table WGCNA significant modules gene list. (XLSX)

S7 Table Tukey's post hoc analysis of the 15 significant WGCNA modules. Signif. codes: ‘***’ 0.001; ‘**’ 0.01; ‘*’ 0.05. Treatments with the same letter are not significantly different.
(XLSX)

S8 Table IPA Functional overrepresentation of Antiquewhite1 module. (XLSX)

S9 Table IPA Functional overrepresentation of Antiquewhite4 module. (XLSX)

S10 Table IPA Functional overrepresentation of Black module. (XLSX)

S11 Table IPA Functional overrepresentation of Blue module. (XLSX)

S12 Table IPA Functional overrepresentation of Brown4 module. (XLSX)

S13 Table IPA Functional overrepresentation of Darkgrey module. (XLSX)

S14 Table IPA Functional overrepresentation of Darkred module. (XLSX)

S15 Table IPA Functional overrepresentation of Firebrick2 module. (XLSX)

S16 Table IPA Functional overrepresentation of Lightcyan module. (XLSX)

S17 Table IPA Functional overrepresentation of Midnightblue module. (XLSX)

S18 Table IPA Functional overrepresentation of Paleturquoise module. (XLSX)

S19 Table IPA Functional overrepresentation of Palevioletred3 module. (XLSX)

S20 Table IPA Functional overrepresentation of Powderblue module. (XLSX)

S21 Table IPA Functional overrepresentation of Salmon module. (XLSX)

S22 Table IPA Functional overrepresentation of Steelblue module. (XLSX)
